# Cholesterol removal from adult skeletal muscle impairs excitation–contraction coupling and aging reduces caveolin-3 and alters the expression of other triadic proteins

**DOI:** 10.3389/fphys.2015.00105

**Published:** 2015-04-10

**Authors:** Genaro Barrientos, Paola Llanos, Jorge Hidalgo, Pura Bolaños, Carlo Caputo, Alexander Riquelme, Gina Sánchez, Andrew F. G. Quest, Cecilia Hidalgo

**Affiliations:** ^1^Physiology and Biophysics Program, Institute of Biomedical Sciences, School of Medicine, University of ChileSantiago, Chile; ^2^Institute for Research in Dental Sciences, Faculty of Dentistry, University of ChileSantiago, Chile; ^3^Centre of Biophysics and Biochemistry, Venezuelan Institute for Scientific ResearchCaracas, Venezuela; ^4^Biomedical Neuroscience Institute, School of Medicine, University of ChileSantiago, Chile; ^5^Pathophysiology Program, Institute of Biomedical Sciences, School of Medicine, University of ChileSantiago, Chile; ^6^Center for Molecular Studies of the Cell, School of Medicine, University of ChileSantiago, Chile; ^7^Laboratory of Cell Communication, Program in Cell and Molecular Biology, Institute of Biomedical Sciences, School of Medicine, University of ChileSantiago, Chile; ^8^Advanced Center for Chronic Diseases and Network for Metabolic Stress Signaling, University of ChileSantiago, Chile

**Keywords:** transverse tubules, Ca^2+^ transients, RyR1, Cav1.1, GAPDH, NADPH oxidase, Na^+^/K^+^-ATPase

## Abstract

Cholesterol and caveolin are integral membrane components that modulate the function/location of many cellular proteins. Skeletal muscle fibers, which have unusually high cholesterol levels in transverse tubules, express the caveolin-3 isoform but its association with transverse tubules remains contentious. Cholesterol removal impairs excitation–contraction (E–C) coupling in amphibian and mammalian fetal skeletal muscle fibers. Here, we show that treating single muscle fibers from adult mice with the cholesterol removing agent methyl-β-cyclodextrin decreased fiber cholesterol by 26%, altered the location pattern of caveolin-3 and of the voltage dependent calcium channel Cav1.1, and suppressed or reduced electrically evoked Ca^2+^ transients without affecting membrane integrity or causing sarcoplasmic reticulum (SR) calcium depletion. We found that transverse tubules from adult muscle and triad fractions that contain ~10% attached transverse tubules, but not SR membranes, contained caveolin-3 and Cav1.1; both proteins partitioned into detergent-resistant membrane fractions highly enriched in cholesterol. Aging entails significant deterioration of skeletal muscle function. We found that triad fractions from aged rats had similar cholesterol and RyR1 protein levels compared to triads from young rats, but had lower caveolin-3 and glyceraldehyde 3-phosphate dehydrogenase and increased Na^+^/K^+^-ATPase protein levels. Both triad fractions had comparable NADPH oxidase (NOX) activity and protein content of NOX2 subunits (p47^phox^ and gp91^phox^), implying that NOX activity does not increase during aging. These findings show that partial cholesterol removal impairs E–C coupling and alters caveolin-3 and Cav1.1 location pattern, and that aging reduces caveolin-3 protein content and modifies the expression of other triadic proteins. We discuss the possible implications of these findings for skeletal muscle function in young and aged animals.

## Introduction

In skeletal muscle, action potentials propagate into the fiber interior through the transverse tubule (T-tubule) system, an intracellular membrane network composed of narrow tubules around 40–85 nm in diameter that originate from deep invaginations of the surface plasma membrane (Melzer et al., 1995; Jayasinghe and Launikonis, 2013). The T-tubule network contains Cav1.1 voltage-dependent Ca^2+^ channels (Anderson et al., 1994)—also known as dihydropyridine receptors (DHPR)—which play a crucial role in skeletal muscle excitation–contraction (E–C) coupling. Skeletal muscle T-tubules form arrangements called triads with the two adjacent terminal cisternae of the sarcoplasmic reticulum (SR) (Franzini-Armstrong, 1972). At the triads, the T-tubule Cav1.1 channels physically interact with the type-1 ryanodine receptor (RyR1), Ca^2+^ release channels present in junctional SR (Marks et al., 1989; Zalk et al., 2007). Muscle depolarization triggers voltage-dependent Cav1.1 conformational changes (Rios et al., 1993; Minarovic and Meszaros, 1998) that elicit RyR1-mediated Ca^2+^ release (Anderson and Meissner, 1995; Fill and Copello, 2002); the ensuing increase in myoplasmic Ca^2+^ concentration ([Ca^2+^]) triggers muscle contraction.

Reports regarding T-tubule lipid composition from vertebrate muscles have revealed some unusual features, characterized by high cholesterol and sphingolipid contents (Lau et al., 1979; Rosemblatt et al., 1981; Hidalgo et al., 1986), which endow T-tubule membranes with an unusually rigid lipid environment similar to that present in thermophilic bacteria (Hidalgo, 1985). The high cholesterol and sphingolipid levels of T-tubule membranes, which are significantly higher than those present in plasma membranes, are comparable to those reported in lipids rafts and caveolae (Smart et al., 1999). Previous studies indicate that treatment of cultured C2C12 muscle cells with the cholesterol-binding drug Amphotericin B considerably reduces the tubular elements connected to the surface (Carozzi et al., 2000). Similarly, treatment of fetal skeletal muscle cells from mice with the cholesterol-lowering agent MβCD decreases surface-connected tubular elements and disorganizes the T-tubule system (Pouvreau et al., 2004). Previous reports indicate that cholesterol removal impairs E–C coupling in amphibian (Launikonis and Stephenson, 2001) and skeletal muscles from fetal mice (Pouvreau et al., 2004). To test the hypothesis that T-tubule cholesterol content is important for E–C coupling in adult muscle, the first aim of the present work was to examine in single skeletal muscle fibers from adult mice the effects of partial cholesterol removal with MβCD on electrically evoked Ca^2+^ transients.

Caveolins are a particular class of membrane proteins that associate directly with cholesterol in lipid rafts and constitute structural components of caveolae. The scaffolding domain of caveolin participates in protein-protein interactions and the regulation of signal transduction events (Williams and Lisanti, 2004). Striated muscle tissue expresses mainly the caveolin-3 protein isoform (Song et al., 1996); yet, few studies have addressed the role of caveolin-3 in striated muscle function. In cardiac muscle cells, caveolin-3 associates with Cav1.2 (L-type) Ca^2+^ channels (Balijepalli et al., 2006). The expression levels of caveolin-3 in skeletal muscle modulate Ca^2+^ currents through Cav1.1 L-type calcium channels and caveolin-3 mutations reduce Cav1.1 currents without altering Cav1.1 expression levels (Weiss et al., 2008), suggesting a role for caveolin-3 in the E–C coupling process. Additionally, recent studies reported direct interaction of caveolin-3 with skeletal muscle RyR1 (Whiteley et al., 2012) and suggested inhibition of mechano-sensitive cation channels by caveolin-3 (Huang et al., 2013). The precise location of caveolin-3 in skeletal muscle fibers remains contentious. Previous studies reported that caveolin-3 is present in the sarcolemma (skeletal muscle surface plasma membrane) associated with the dystrophin complex (Song et al., 1996), and that during muscle differentiation caveolin-3 associates with developing T-tubules but is absent from mature T-tubules (Parton et al., 1997). A later study in soleus muscle from adult rats, however, reported that while caveolin-3 occurs at the highest density on the plasma membrane, it is also present in T-tubules (Ralston and Ploug, 1999). Caveolin-3 knockout mice present T-tubule system abnormalities (Galbiati et al., 2001), consistent with a T-tubule location, and also exhibit muscle degeneration (Hagiwara et al., 2000). A more recent study reported the presence of caveolin-3 in isolated SR vesicles (Li et al., 2006), albeit the low cholesterol content of these membranes (Lau et al., 1979; Rosemblatt et al., 1981) makes this location unlikely. Hence, to test the hypothesis that in adult muscle fibers caveolin-3 is present in the T-tubules, the second aim of this work was to study caveolin-3 location in adult mammalian skeletal muscle.

Aging entails significant deterioration of skeletal muscle function (Miller et al., 2014). Among other changes, a decreased intracellular Ca^2+^ peak associates with the reduction in muscle force observed during aging (Booth et al., 1994; Wang et al., 2000). Age-related changes in T-tubule protein and lipid composition might contribute to the observed defects in skeletal muscle function. In fact, previous work suggested that aged rats have uncoupled Cav1.1-RyR1 channels (Renganathan et al., 1997) and display a 60% reduction in Cav1.1 protein levels (O'connell et al., 2008). No information is available, however, on changes in T-tubule cholesterol and caveolin-3 levels during aging. Therefore, to test the hypothesis that aging brings about changes in transverse tubule components, which might contribute to aged-related defective skeletal muscle function, the third aim of the present study was to investigate if aging modifies T-tubule cholesterol levels and to compare, in addition, the levels of key triadic proteins including caveolin-3 in young and aged rats. For this purpose, we used triad-enriched membrane fractions obtained from adult skeletal muscle that contain ~10% attached T-tubules (Hidalgo et al., 1993), and which after isolation maintain the morphological features found in intact muscle (Wagenknecht et al., 2002). In particular, we investigated if caveolin-3 levels change in aged animals since alterations in the levels of this scaffolding protein may result in ion channel dysregulation, among other effects.

Our results show that partial cholesterol extraction from isolated single skeletal fibers impaired E–C coupling and altered Cav1.1 and caveolin-3 distribution. We also found caveolin-3 in T-tubules and T-tubule-containing triad fractions but not in SR membranes, and in cholesterol-enriched detergent-resistant membrane (DRM) fractions from T-tubules or triads. We observed significantly decreased caveolin-3 and glyceraldehyde 3-phosphate dehydrogenase (GAPDH) and increased Na^+^/K^+^-ATPase levels in triads from aged rats, but we did not detect changes in cholesterol and RyR1 protein levels. We discuss the possible implications of these findings for skeletal muscle E–C coupling in young and aged animals.

## Materials and methods

### Animals

Male New Zealand white rabbits (6-month-old), Balb/C mice (8-week-old) and Sprague Dawley rats (3 or 24 months-old) were obtained from the Animal Facility at the Faculty of Medicine, Universidad de Chile. Room temperature was kept constant at 21°C, and light was maintained on a 12:12 h light-dark cycle. Mice were sacrificed by quick cervical dislocation. Rabbits and rats were euthanized by intraperitoneal overdose of sodium pentobarbital (100 mg/kg). All experiments were carried out following the guidelines provided by National Institutes of Health (USA) and the regulations for the Care and Use of Animals for Scientific Purposes; the Bioethics Committee of the Faculty of Medicine, Universidad de Chile approved all animal procedures performed in this work.

### Materials

All reagents used were of analytical grade. Protease inhibitors (leupeptin, pepstatin A, benzamidine, and phenylmethylsulfonyl fluoride) were from Sigma-Aldrich (St. Louis, MO), MagFluo-4 AM and Fluo-4 AM were from Invitrogen (Carlsbad, CA), and Matrigel was from BD Biosciences (San Jose, CA). Paraformaldehyde was from Electron Microscopy Science (Hatfield, PA), Dulbecco's modified Eagle's medium supplemented with 10% fetal bovine serum was from Invitrogen (Carlsbad, CA) and was supplemented with 0.1 mg/ml penicillin–streptomycin from Sigma-Aldrich (St. Louis, MO). The BCA protein assay kit was from Pierce Biotechnology, Inc. (Rockford, IL), commercial BSA was from Sigma-Aldrich (St. Louis, MO) and Dako anti-fading reagent was from Dako (Denmark). Horseradish peroxidase-conjugated anti-IgG (anti-mouse or anti-rabbit) were from Santa Cruz Biotechnology, Inc. (Santa Cruz, CA). Antibodies against the α1s subunit of Cav1.1 were from Affinity BioReagents (Golden, CO; mouse monoclonal) or from Santa Cruz Biotechnology, Inc. (Santa Cruz, CA; rabbit polyclonal). Alexa Fluor-488 anti-mouse and Alexa Fluor-633 anti-rabbit antibodies were from Invitrogen (Carlsbad, CA). Caveolin-3 antibodies were from BD Biosciences (San Jose, CA) or Santa Cruz Biotechnology (Santa Cruz, CA), gp91^phox^ and p47^phox^ antibodies were from Santa Cruz Biotechnology (Santa Cruz, CA), GAPDH antibody was from Sigma-Aldrich (St. Louis, MO), RyR1 (34C) and Na^+^/K^+^-ATPase (α6F) antibodies were from Developmental Studies Hybridoma Bank http://dshb.biology.uiowa.edu/. The Amplex Red Cholesterol Assay kit was from Invitrogen (Carlsbad, CA), the total cholesterol assay kit was from Labtest (Sao Paulo, Brazil) and the ECL kit was from Thermo Fisher Scientific Inc. (Rockford, IL).

### Fiber isolation from adult skeletal muscle

Flexor digitorum brevis (FDB) muscles were dissected from 8 week-old mice and single intact myofibers were isolated enzymatically as described (Carroll et al., 1995; Barrientos et al., 2009). Isolated fibers were plated on Matrigel-coated coverslips and maintained in Dulbecco's modified Eagle's medium supplemented with 10% fetal bovine serum and 0.1 mg/ml penicillin–streptomycin. Fibers were kept overnight in an incubator under 5% CO_2_, and experiments were conducted within 12–24 h of plating.

### Immunofluorescence

Dissociated single fibers from adult mice FDB muscles were plated on 35 mm coverslips coated with Matrigel. After washing with PBS, fibers were fixed by incubation for 10 min at room temperature with PBS supplemented with 4% paraformaldehyde. Next, fibers were rinsed with PBS, permeabilized with 0.1% TritonX-100 in PBS, rinsed with PBS and blocked for 1 h with PBS-1% BSA at room temperature. Fibers were incubated overnight with polyclonal rabbit antibodies against caveolin-3 (1:100, Santa Cruz Biotechnology; Santa Cruz, CA,) and Cav1.1 (1:100). Fibers were washed and incubated 1 h with Alexa Fluor-488 anti-mouse and Alexa Fluor-633 anti-rabbit antibodies. Samples treated with Dako anti-fading reagent were stored at 4°C until use.

### Fluorescence recording and field stimulation

All records were collected from single fibers bathed in mammalian Ringer solution (mM: 145 NaCl, 2.5 KCl, 1.0 MgSO_4_, 2.5 CaCl_2_, 10 glucose, 10 Hepes/Tris, pH 7.4). Fibers were incubated for 40 min at room temperature with 10 μM MagFluo-4 AM in mammalian Ringer solution containing 0.01% pluronic acid. Alternatively, fibers were loaded at room temperature for 40 min in mammalian Ringer plus 0.01% pluronic acid with the higher affinity Ca^2+^ dye Fluo-4 AM (10 μM). Fibers adhering spontaneously to the glass bottom of the experimental chamber were selected for fluorescence recording. The experimental chamber was mounted on the stage of an inverted Nikon Diaphot TMD microscope equipped for epifluorescence. Fibers were illuminated with a xenon lamp (100 W) only during recording to avoid dye photobleaching. The characteristic wavelengths of filter combinations (excitation/dichroic/barrier) were (nm) 450–490/510/520. Light signals were collected from a spot of approximately 12 μm diameter, with a photomultiplier connected to a Nikon P1 amplifier. This procedure allowed recording from several fibers within the microscope field (Calderon et al., 2011).

Field stimulation leading to intracellular Ca^2+^ transients was elicited by applying supra-threshold rectangular current pulses (0.2–0.4 ms duration) through two platinum plate electrodes placed on either side along the experimental chamber. The amplifier output was connected to an Axon Instruments TL1 DMA interface. Data were acquired and analyzed using the Axon Instruments pCLAMP 6 software. Fluorescence values are expressed as Δ*F/F*_rest_[(*F* − *F*_rest_)/*F*_rest_], where *F*_rest_ correspond to the basal fluorescence recorded before stimulation (Capote et al., 2005).

### Membrane fractions

Triad-enriched fractions (hereafter referred to as triads) containing on average 10% attached T-tubules were isolated from rabbit or rat muscle as described previously (Hidalgo et al., 1993). T-tubule membranes, heavy sarcoplasmic reticulum (HRS) and light reticulum (LSR) from skeletal muscle were isolated as previously described (Rosemblatt et al., 1981), with some modifications. Briefly, 100 g of back muscles from rabbit or 15 g from the back and hind limb skeletal muscles from male rats were homogenized in four volumes of buffer A (mM: 100 KCl. 20 MOPS-Tris, pH 7.0). The suspension was centrifuged at 10,000 × g and the sediment was homogenized in buffer A, adjusted to 0.6 M KCl by solid salt addition and centrifuged for 1 h at 100,000 × g. The pellets were resuspended in buffer A containing a combination of protease inhibitors (1 μg/ml leupeptin, 1 μg/ml pepstatin, 0.4 mM benzamidine 1 mM phenylmethanesulfonylfluoride), and sedimented at 100,000 × g. The pellets were resuspended in sucrose buffer (buffer B; mM: 300 sucrose, 20 MOPS-Tris, pH 7.0, plus 1 μg/ml leupeptin, 1 μg/ml pepstatin, 0.4 mM benzamidine) and centrifuged at 100,000 × g. The resulting pellets were resuspended in buffer B, loaded on a discontinuous sucrose gradient (45, 35, 27.5, and 25% w/v) and centrifuged at 100,000 × g overnight. T-tubules were collected from the 25/27.5% interface. The HSR and LSR fractions were collected from the 35/45 and the 27.5/35% interfaces, respectively. Fractions were resuspended in buffer B, centrifuged for 30 min at 100,000 × g and the pellets were resuspended in a minimum volume of buffer B, frozen in liquid N_2_ and stored at −80°C until use.

Junctional T-tubule fractions were prepared from triads as described (Horgan and Kuypers, 1987). Briefly, the triad fraction was loaded on top of an ion-free sucrose gradient (1.4–0.74 M) and centrifuged 16 h at 100,000 × g. This treatment separates the junctional T-tubule membranes from the triads and yields a light fraction containing junctional T-tubules. This fraction was collected, diluted in 20 mM MOPS/Tris, pH 7.2 supplemented with 1 μg/ml leupeptin, 1 μg/ml pepstatin, 0.4 mM benzamidine, and centrifuged for 1 h at 100,000 × g. The pellet, resuspended in a small volume of buffer B containing 1 μg/ml leupeptin, 1 μg/ml pepstatin, was stored at −80°C until use.

### Detergent-resistant membrane (DRM) fractions

DRM fractions were prepared from triads and T-tubule membranes as described (Brown and Waneck, 1992; Sargiacomo et al., 1993) with minor modifications. Briefly, membrane fractions were resuspended and mixed in 2 ml (final volume) of Mes buffer saline (MBS, in mM: 150 NaCl, 25 Mes/NaOH, pH 6.5) containing 1% Triton X-100 and protease inhibitors (1 μg/ml leupeptin, 1 μg/ml pepstatin, 1 mM phenylmethanesulfonylfluoride, 0.4 mM benzamidine) and incubated for 15 min at 4°C. The suspensions, adjusted to 40% (w/v) sucrose by adding 2 ml of ice-cold 80% sucrose (w/v) in MBS, were placed at the bottom of an ultracentrifuge tube: two equal volume layers of 30 and 5% sucrose were added on top, and the gradients were centrifuged at 100,000 × g for 20 h. The light opalescent band confined to the 5–30% interface was collected, diluted three times with MBS and centrifuged at 100,000 × g for 1 h. The fractions thus obtained were resuspended in buffer B, frozen in liquid nitrogen and stored at −80°C until use.

### SDS/PAGE and Western blot analysis

Membrane preparations were heat-denatured in SDS-containing sample buffer with reducing agents and loaded onto Tris-glycine 4–12% acrylamide gradient SDS-containing gels. Following electrophoresis at 100 V, proteins were transferred to nitrocellulose membranes at 100 V for 1 h. Membranes, blocked with 5% non-fat dry milk in Tris-saline buffer (mM: 140 NaCl, 20 Tris-HCl, pH 7.6) plus 0.2% Tween 20 were probed with specific antibodies directed against T-tubule or SR proteins. Horseradish peroxidase-conjugated anti-mouse or anti-rabbit IgG were used as secondary antibodies. Immunoblots were developed using an ECL kit and automated image capture was performed with the ChemiDoc system (Bio-Rad, Hercules, CA). All blot quantifications involved measuring band intensities; the amount of protein loaded in each lane was checked by Coomassie blue staining.

### Other procedures

NOX activity was determined as described previously (Sanchez et al., 2008). Protein concentration was determined by the BCA protein assay using commercial BSA as a standard. Cholesterol content was determined using the colorimetric total cholesterol kit or the fluorescence Amplex Red Cholesterol Assay kit, as detailed in the text. Electron microscopy (EM) analysis of DRM fractions was performed as described (Badizadegan et al., 2000).

### Statistical analysis

To detect significant differences between two groups, statistical analysis of data, presented as Mean ± SE, was determined with the Student's *t*-test (two-tailed); a value of p < 0.05 was considered statistically significant.

## Results

### Membrane cholesterol reduction impairs skeletal muscle E–C coupling

**Figure 1:**
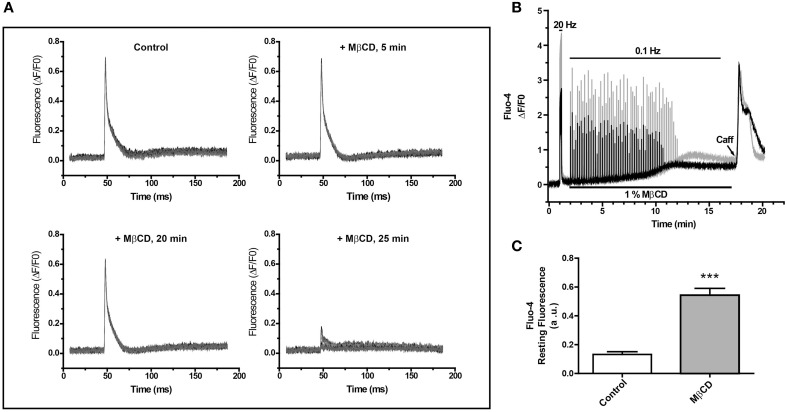
**Perfusion of single fibers from adult skeletal muscle with MβCD abolishes calcium transients and increases resting calcium levels**. **(A)** FDB fibers were loaded with the low affinity Ca^2+^ sensor Mag-Fluo-4 and Ca^2+^ transients were elicited by electrical field stimulation. Fluorescence was evaluated before and after the addition of 1% MβCD. Each panel shows 10 transients, collected at 10 s intervals at the times indicated in the figure. **(B)** Ca^2+^ transients were elicited by electric field stimulation of FDB fibers loaded with Fluo-4, a high affinity Ca^2+^ probe. The traces show the fluorescence of two fibers and their responses to field stimulation at 20 or 0.1 Hz. The continuous line at the top represents the period of electric field stimulation, the lower continuous line indicates the period of perfusion with MβCD. The arrowhead indicates addition of 10 mM caffeine. **(C)** Average Fluo-4 resting fluorescence collected 20 min after initiation fluorescence recording in control (*n* = 17) or MβCD-treated (*n* = 25) fibers.

To study whether decreasing the high T-tubule cholesterol levels affects the E–C coupling process, we measured electrically induced Ca^2+^ transients in FDB fibers from adult mice before and after addition of the cholesterol-lowering agent MβCD. The representative experiment illustrated in Figure 1A shows Ca^2+^ transients recorded in fibers loaded with the low-affinity Ca^2+^ probe MagFluo-4 before or 5, 20, and 25 min after perfusion with 1% MβCD, which abolished Ca^2+^ transients after 25 min. This treatment impaired Ca^2+^ transients in all fibers tested, isolated from six different animals. Of note, MβCD abolished Ca^2+^ transients in 26 of the 30 fibers tested and in the remaining 4 fibers it significantly reduced Ca^2+^ transient amplitude by 51.3 ± 27.6 (%).

To assay whether MβCD treatment modified resting myoplasmic free [Ca^2+^], which did not display apparent changes in fibers loaded with the low affinity Ca^2+^ probe MagFluo-4 (Figure 1A), we loaded fibers with Fluo-4, a high affinity fluorescent Ca^2+^ probe, and determined Ca^2+^ transients elicited by electrical field stimulation. The representative experiment illustrated in Figure 1B shows that initial stimulation at 20 Hz elicited significant Ca^2+^ transients in two fibers recorded in parallel. After perfusion with 1% MβCD, both fibers displayed for about 8 min Ca^2+^ transients of similar magnitudes in response to stimulation at 0.1 Hz, after which time the response declined abruptly and vanished within 2 min (Figure 1B). The same results were obtained in 26 fibers from five different mice treated with MβCD. In contrast, and in agreement with previous findings (Barrientos et al., 2009), control fibers (18 fibers from 5 mice) loaded with Fluo-4 AM in mammalian Ringer solution containing 0.01% pluronic acid, responded with Ca^2+^ transients of equal magnitude for at least 30 min when stimulated at 0.1 Hz (not shown).

Perfusion with 1% MβCD produced an increase in resting fluorescence coincident with the decline in Ca^2+^ transient amplitude, as evidenced from the parallel records from two fibers illustrated in Figure 1B; the Fluo-4 fluorescence increase reached a plateau 9–11 min after MβCD addition. Average fluorescence values recorded 20 min after MβCD addition showed a small but significant baseline fluorescence increase in MβCD-treated fibers (*n* = 25) relative to the controls (*n* = 17) (Figure 1C). This increase was observed at levels far from probe saturation, as indicated by the considerably higher fluorescence increase produced by the initial stimulation at 20 Hz. From this higher value and the K*_d_* (345 μM) for Fluo-4, we estimate that the net increase in resting [Ca^2+^] was < 100 nM. Perfusion with 10 mM caffeine after MβCD removal, when resting [Ca^2+^] reached a plateau, produced a transient and significant increase in fluorescence (Figure 1B). The caffeine-stimulated fluorescence increase was observed in a large fraction (23/26) of MβCD-treated fibers and in all (18/18) control fibers.

These combined results indicate that perfusion with 1% MβCD, which decreases cholesterol levels by 26% in FDB fibers from adult muscle (Table 1), abolishes electrically evoked Ca^2+^ transients without significantly perturbing the membrane permeability barrier, as indicated by the lack of massive increase in myoplasmic [Ca^2+^]. Additionally, the stimulatory effects of caffeine strongly suggest that the lack of response to electrical stimulation in MβCD-treated fibers is not due to SR depletion and presumably reflects impaired E–C coupling.

**Table 1:** **Cholesterol content in mice skeletal fibers and membrane fractions**.

**Assayed sample**	**Cholesterol (μg/mg protein)**
**SKELETAL FDB FIBERS**					
A. Control	17.6 ± 1.2 (4)
B. MβCD-treated	13.1 ± 1.4 (4)
**MEMBRANE FRACTIONS**					
C. T-tubules (rabbit)	271.6 ± 14.8 (3)
D. DRM from T-tubules (rabbit)	1233.0 ± 69.55 (4)
Ratio D/C	4.5
E. Triads (rabbit)	41.5 ± 2.3 (4)
F. DRM from Triads (rabbit)	184.3 ± 4.9 (4)
Ratio F/E	4.4
G. Plasma membrane (Ortegren et al., 2004)	143
H. Caveolae (Ortegren et al., 2004)	400.5 ± 21
Ratio H/G	2.8

*Total cholesterol content was measured, with the exception of the DRM fractions, with a commercial colorimetric kit; cholesterol content of DRM fractions was determined with the Amplex Red kit (for details see Materials and Methods). FDB cholesterol content was measured in sonicated whole fiber homogenates. Data represent Mean ± SE; the number of different preparation assayed is given in parenthesis*.

### Caveolin-3 associates with T-tubule and triad membranes and is absent from SR fractions

Previous reports suggest a role for caveolin-3 in skeletal muscle E–C coupling (Weiss et al., 2008; Whiteley et al., 2012), which, as reported here, is sensitive to cholesterol removal from adult fibers. The caveolin proteins bind directly to cholesterol (Murata et al., 1995) and their presence in membranes correlates directly with membrane cholesterol levels (Toselli et al., 2005); however, the location of caveolin-3 in T-tubule membranes remains debatable. Accordingly, we tested here the location of caveolin-3 by immunohistochemistry of adult fibers from mice and by Western blot analysis of membrane fractions isolated from rabbit or rat skeletal muscle.

**Figure 2:**
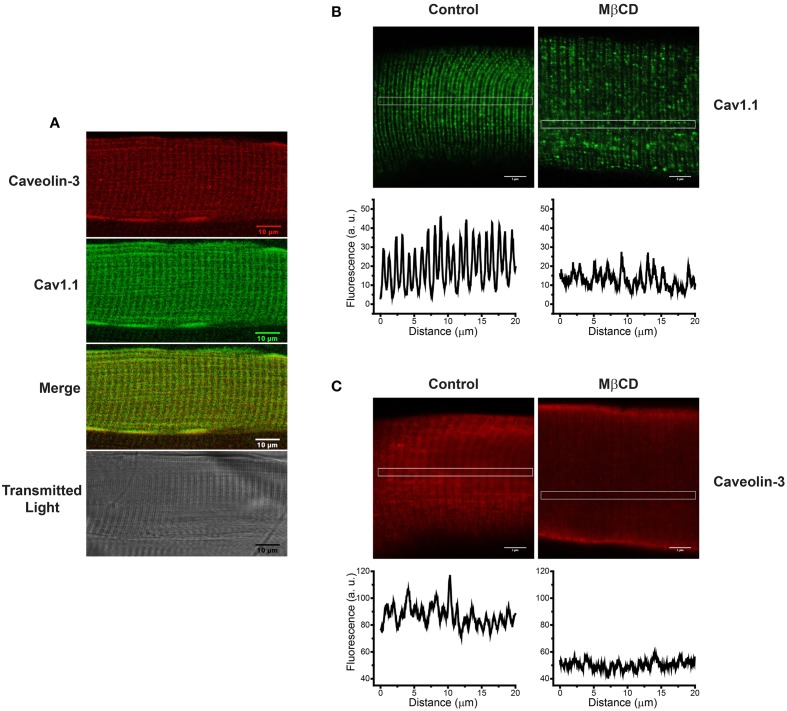
**Caveolin-3 is associated to T-tubules membranes in intact FDB fibers from adult skeletal muscle; treatment with MβCD alters the distribution of Cav1.1 and caveolin-3**. **(A)** Immunohistochemistry of a single FDB fiber isolated from skeletal muscle of adult mice. The top panel shows caveolin-3 in red, the central panel shows Cav1.1 in green, the panel with the merged image shows the superposition of both fluorescence signals, and the lower panel shows the transmitted light image. **(B)** Distribution of Cav1.1 (green) in control (left panel) and fibers treated with 1% MβCD (right panel). The graphs under each image illustrate the horizontal fluorescence profiles collected from the rectangular region of interest (ROI) indicated in the images. **(C)** Distribution of caveolin-3 (red) in control (left panel) and MβCD-treated fibers (right panel). The graphs under each image illustrate the horizontal fluorescence profiles collected from the rectangular ROI indicated in each image. Scale bar: 5 μm.

As illustrated in Figure 2A, caveolin-3 co-localizes with Cav1.1 in FDB fibers from adult mice skeletal muscle. Treatment of with 1% MβCD, which reduced cholesterol content by 26% (Table 1), produced a significant change in the distribution of both Cav1.1 (Figure 2B) and caveolin-3 (Figure 2C). After MβCD treatment, both proteins exhibited a decrease in the characteristic banding pattern displayed by control fibers, suggesting that partial reduction of fiber cholesterol content alters the typical array of Cav1.1 in the T-tubule membrane (Figure 2B) and reduces the association of caveolin-3 with T-tubules (Figure 2C). Similar results were obtained in 3–9 fiber preparations from each mouse, out of a total of four mice.

**Figure 3:**
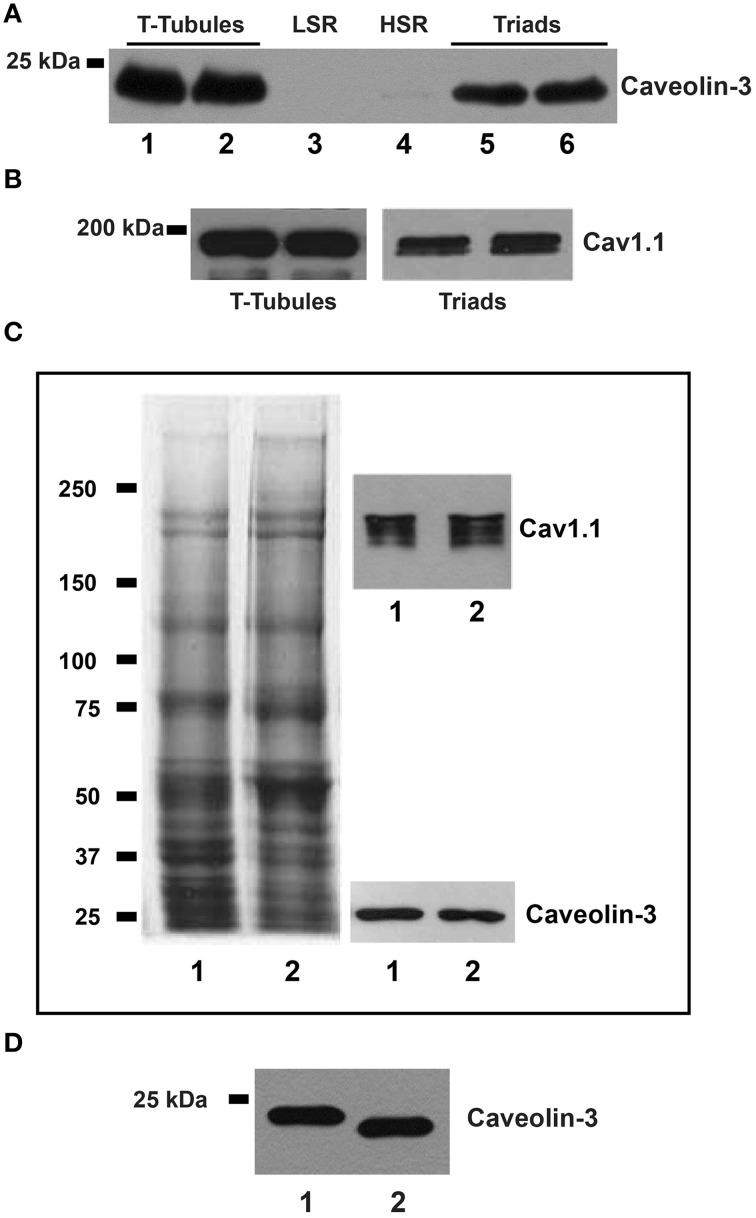
**Caveolin-3 is associated to T-tubules and triad fractions from adult skeletal muscle**. **(A)** The representative upper blot shows membrane fractions from rabbit skeletal muscle, resolved by SDS/PAGE, transferred and tested with caveolin-3 or Cav1.1 antibodies. Each lane was loaded with 20 μg of protein. T-tubules, lanes 1 and 2; LSR, lane 3; HSR, lane 4; triads, lanes 5 and 6. **(B)** Separate blot showing that T-tubule and triad fractions contain Cav1.1. **(C)** The left panel shows a Coomassie blue stained gel; lane 1: T-tubules (20 μg), lane 2: junctional T-tubules (20 μg). The right panel shows the corresponding Western blots for caveolin-3 and Cav1.1, in the same samples loaded on the left panel. **(D)** Western blot of triad fractions; each lane was loaded with 20 μg of protein. Lane 1: triads from rabbit; lane 2: triads from rat.

The representative Western blot illustrated in Figure 3A shows that caveolin-3 is present in T-tubules (lanes 1–2) and triads (lanes 5–6) isolated from rabbit skeletal muscle, but is absent from the LSR (lane 3) and HSR (lane 4) fractions, which are highly enriched in Ca^2+^-ATPase and RyR1/calsequestrin, respectively (not shown). We obtained similar results in 3 independent preparations. The separate blots shown in Figure 3B confirm that T-tubule and triad fractions contain Cav1.1. These findings show unambiguously that caveolin-3 is present in T-tubules and T-tubule-containing triads but is absent from SR membranes. To further assay the specific association of caveolin-3 with T-tubule membranes, we dissociated triads isolated from rabbit muscle to generate a light fraction enriched in junctional T-tubule membranes (Horgan and Kuypers, 1987). The representative whole gel (stained with Coomassie blue) illustrated in Figure 3C (left) shows that the protein profile of T-tubules isolated from whole rabbit skeletal muscle (lane 1) is very similar to that of the junctional T-tubule fraction obtained from dissociated triads (lane 2). The respective Western blots shown at right in Figure 3C also indicate that both T-tubule fractions contain comparable caveolin-3 and Cav1.1 protein levels. Similar results were obtained in two different preparations. We also found caveolin-3 in triads from rat (Figure 3D, lane 2) that at equal protein loads migrated slightly faster but displayed similar band density as the caveolin-3 band present in triads from rabbit skeletal muscle (Figure 3D, lane 1). Junctional T-tubules isolated from rat triads also contained significant levels of caveolin-3 (data not shown).

### Detergent resistant membranes (DRM) from skeletal muscle contain high cholesterol levels, caveolin-3 and Cav1.1

**Figure 4:**
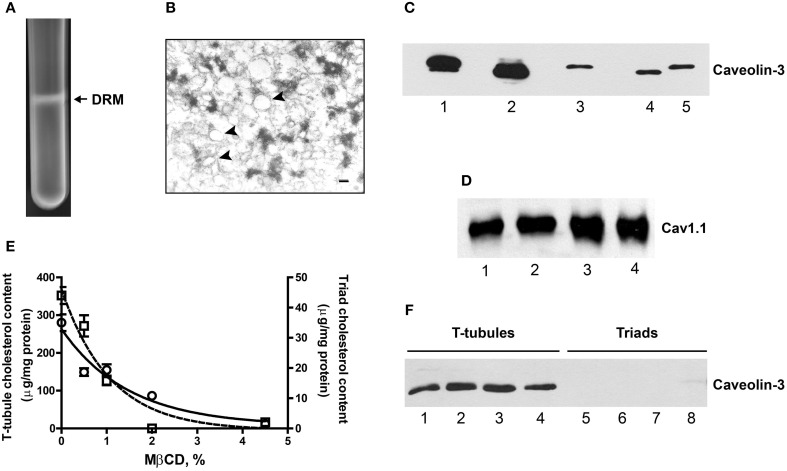
**DRM fractions from triads are enriched in caveolin-3 and contain Cav1.1. (A)** Photograph of a centrifuge tube after 16 h of centrifugation of a sucrose gradient loaded with Triton X-100-treated triads, showing the appearance of an opalescent band containing the DRM fraction (for details, see the Experimental Procedures Section). **(B)** Representative EM photograph of a DRM fraction from triads. Unilamellar vesicles appear associated to electron dense material (arrows). Magnification: 150,000×; calibration bar, 20 nm. **(C)** Representative Western blot revealed with caveolin-3 antibodies. Lanes 1 and 2: 15 μg DRM isolated from triads from rabbit or rat, respectively. Lane 3: 15 μg of junctional T-tubules from triads from rabbit. Lanes 4 and 5: 20 μg, triads from rat or rabbit, respectively. Spaces between thes lanes correspond to empty lanes. **(D)** Western blot revealed with Cav1.1 antibodies. Lanes 1 and 2: 13 μg, DRM from triads from rabbit. Lanes 3 and 4: 6 μg, DRM from T-tubules from rabbit. **(E)** T-tubule and triads fractions from rabbit were treated with increasing MβCD concentrations, pelleted at 100,000 × g and the membrane cholesterol and soluble caveolin-3 were evaluated (for details, see Experimental Procedures Section). The graph shows the cholesterol content of MβCD-treated T-tubules (left y-axis) or triads (right y-axis). **(F)** Western blot showing caveolin-3 protein content in the supernatants of MβCD-treated T-tubules (lanes 1–4) or triads (lanes 5–8). Similar results were obtained in two different experiments.

Incubation of T-tubules or triads with 1% Triton [X-100] (for details, see Experimental Procedures) yielded a light DRM fraction at the 5/30% sucrose interface. This fraction was visible to the naked eye, as illustrated in Figure 4A for the DRM fraction isolated from rabbit T-tubules; the representative electron micrograph of the DRM isolated from triads (rabbit) illustrated in Figure 4B revealed the presence of unilamellar vesicles (arrowheads) associated with electron-dense material.

DRM fractions isolated from triads migrated at a higher sucrose density (25–28% sucrose, w/v) than DRM fractions from T-tubules (16–18% sucrose, w/v). Both fractions had higher cholesterol contents than the corresponding initial fractions, albeit DRM fractions from triads had lower cholesterol levels than DRM fractions from T-tubules (Table 1). For comparison, the isolated caveolae and plasma membrane cholesterol levels (400 and 143 μg/mg protein, respectively) reported in the literature (Ortegren et al., 2004) yield a caveolae/plasma membrane cholesterol ratio of 2.8. Our results yield almost two-fold higher values for the ratios between the cholesterol contents of DRM from T-tubules or triads relative to the original membranes (Table 1). Immunoblot analysis (Figure 4C) revealed a significant enrichment in caveolin-3 in DRM fractions from triads (rabbit, lane 1; rat, lane 2) when compared to the original triad fractions (rat, lane 4; rabbit, lane 5) or to the junctional T-tubule membranes from rabbit (lane 3). As illustrated in Figure 4D, DRM fractions from triads (lanes 1–2) or T-tubules (lanes 3–4) also contained significant levels of the Cav1.1 protein.

To further test the association of caveolin-3 with cholesterol, we treated T-tubules or triads from rabbit with increasing concentrations of MβCD, centrifuged the fractions at 100,000 × g for 5 min and determined cholesterol and caveolin-3 protein contents in the resulting pellets and supernatants, respectively. As illustrated in Figure 4E, treatment with MβCD produced a concentration-dependent decrease in the cholesterol content of pellets from T-tubules (open circles) and triads (open squares). Analysis of caveolin-3 content in the respective supernatants of MβCD-treated fractions (Figure 4F) revealed that the soluble fractions from T-tubules contained caveolin-3 (lanes 1–4) whereas, the soluble fractions from triads lacked this protein (lanes 5–8).

### Triads from aged rats contain decreased protein levels of caveolin-3 and GAPDH

**Figure 5:**
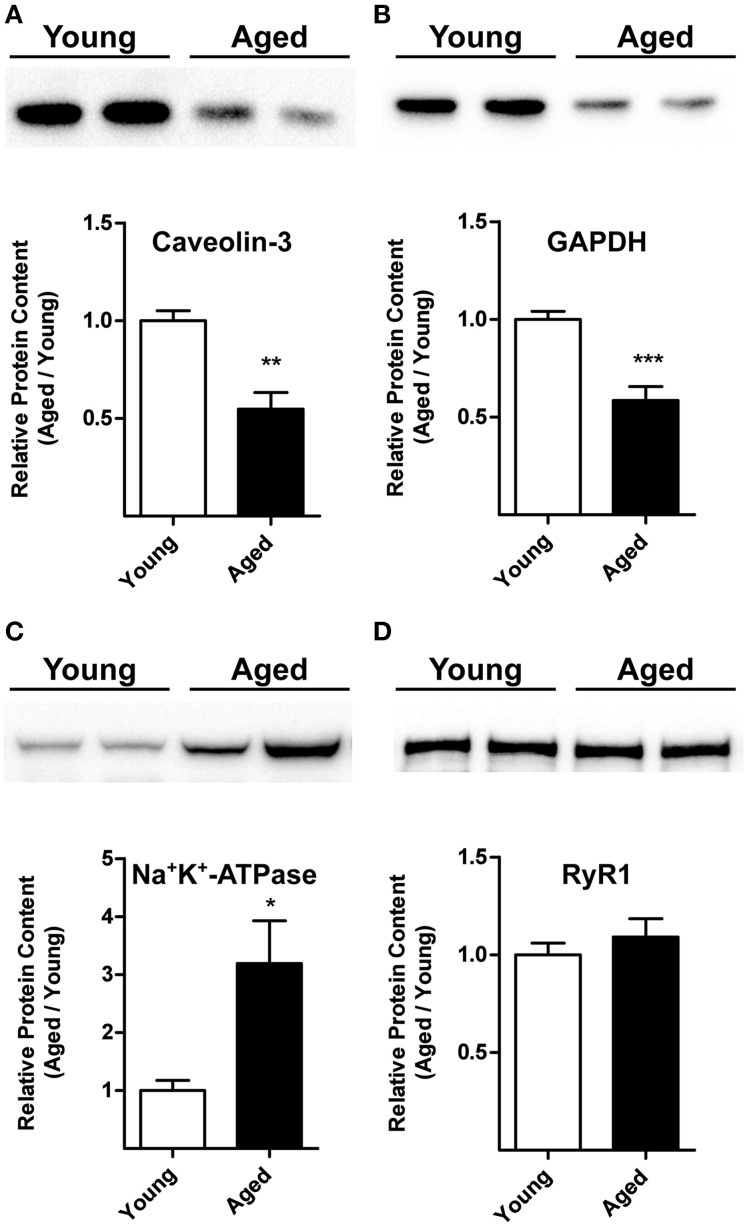
**Caveolin-3, GAPDH, Na^+^/K^+^-ATPase, and RyR1 protein contents in triads from young or aged rats. (A,B)** The top panels illustrate representative Western blots. Each lane was loaded with 10 μg of protein assayed for caveolin-3 and GAPDH, respectively. **(C)** The top panel illustrates a representative Western blot for Na^+^/K^+^-ATPase; each lane was loaded with 30 μg of protein. **(D)** The top panel illustrates a representative Western blot for RyR1; each lane was loaded with 5 μg of protein. Each lane in the blots illustrated in **(A–D)** corresponds to triad fractions from different rats. The lower graphs in **(A**–**D)** present values (Mean ± SE) collected from triad fractions from 6 young and 6 aged rats. ^*^p < 0.05; ^**^p < 0.01; ^***^p < 0.001.

The representative Western blot illustrated in Figure 5A shows that triads from aged rats (24-month-old) contain significantly lower caveolin-3 protein contents than triads from young (3-month-old) rats. Likewise, triads from aged rats contain lower GAPDH levels (Figure 5B). Results from 6 aged and 6 young rats indicate both reductions are statistically significant (Figures 5A,B).

### Triads from aged rats contain increased Na^+^/K^+^-ATPase α1 subunit protein levels

The representative Western blot illustrated in Figure 5C indicates that triads from aged rats contain significant higher Na^+^/K^+^-ATPase α1 subunit levels than triads from young rats. Results from 6 aged and 6 young rats indicate that these differences are statistically significant (Figure 5C).

### Triad RyR1 protein levels and cholesterol content do not change with age

Triads from young or aged rats contained comparable RyR1 protein levels, as shown in the representative Western blot illustrated in Figure 5D and by the average results from 6 young and 6 aged animals summarized in the graph shown in Figure 5D. We obtained inconclusive results regarding Cav1.1 protein levels. Triad fractions from four aged rats displayed similar Cav1.1 protein contents as young rats, but triads from two aged rats had significantly higher Cav1.1 protein levels. All six triad fractions assayed had comparable RyR1 and cholesterol contents (see below), presumably ruling out significant differences in T-tubule content among these fractions.

Triads from aged rats contain on average 51.3 ± 2.6 μg cholesterol/mg protein (*n* = 6; Mean ± SE); these values are not significantly different from the levels determined in triads from young rats: 53.2 ± 3.5 μg cholesterol/mg protein (*n* = 6; Mean ± SE).

### Determination of NOX subunits (gp91^phox^ and p47^phox^) protein contents and NOX activity in young and aged rats

**Figure 6:**
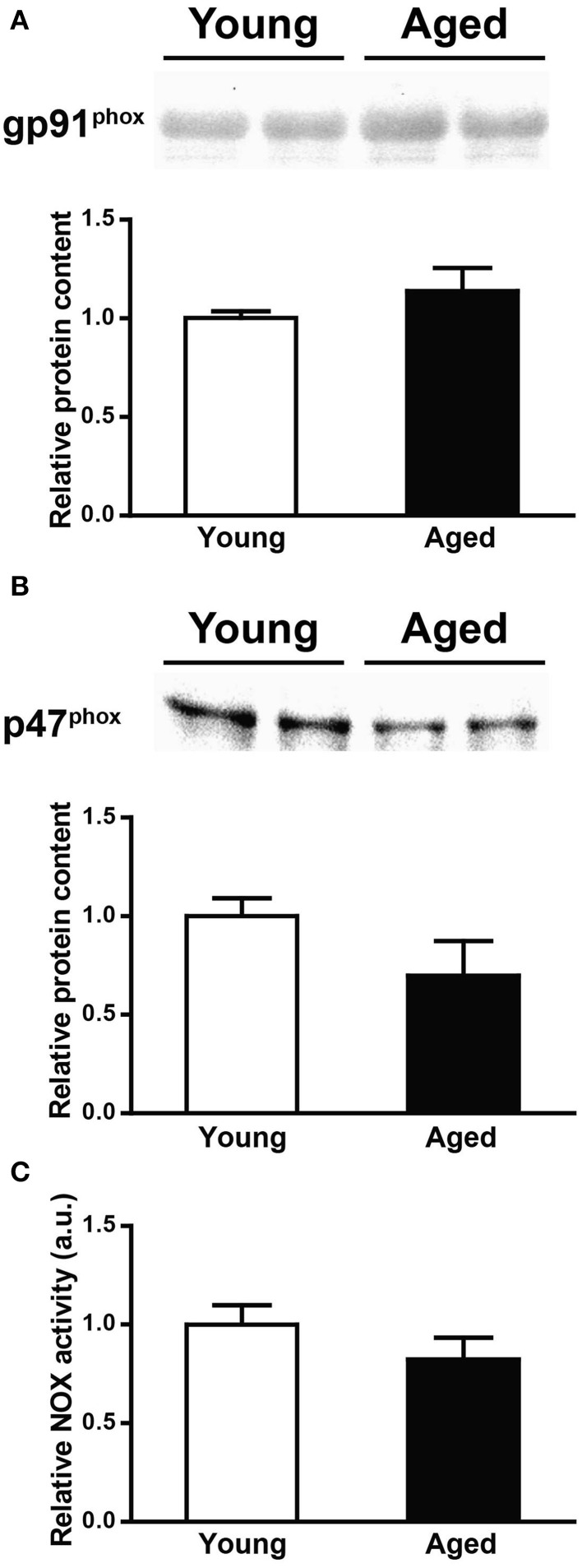
**gp91^phox^ and p47^phox^ protein levels and NOX activity in triads from young or aged rats. (A)** The top panel illustrates a representative Western blot for gp91^phox^; each lane was loaded with 40 μg of protein of triad fraction from different rats. **(B)** The top panel illustrates a representative Western blot for p47^phox^; each lane was loaded with 15 μg of protein of triad fraction from different rats. The lower graphs in **(A**,**B)** summarize values (Mean ± SE) collected from triad fractions from 6 young and 6 aged rats. **(C)** NOX activity of triad fractions from young or aged rats; values (Mean ± SE) correspond to 6 young and 6 aged rats.

As illustrated by the representative Western blot and the graph illustrated in Figure 6A, gp91^phox^ protein levels did not change with age. The same observation applies to p47^phox^ protein levels (Figure 6B); in this case, we observed a tendency toward a decrease with age, which was not statistically significant. In concordance with the lack of change of both gp91^phox^ and p47^phox^ protein levels, we found similar NOX activities in triads from young and aged rats (Figure 6C).

To summarize, the above results show that aging decreased caveolin-3 and GAPDH protein levels, increased the levels of the Na^+^/K^+^-ATPase protein, but did not modify either RyR1, gp91^phox^ and p47^phox^ protein levels or NOX activity.

## Discussion

In this work, we addressed three related aspects. First, we tested the effects of the cholesterol-lowering agent MβCD on Ca^2+^ transients elicited by electrical field stimulation of skeletal fibers from adult mice, and found that MβCD reduced Ca^2+^ transients without disrupting fiber membrane integrity or emptying the SR of its Ca^2+^ content. Second, using several experimental strategies we determined unambiguously that caveolin-3, a cholesterol-associated protein, is present in T-tubules but not in HSR and LSR vesicles. Additionally, we found that treatment with MβCD altered the distribution of both Cav1.1 and caveolin-3 in FDB fibers from adult mice. Third, we found decreased caveolin-3 and GAPDH and increased Na^+^/K^+^-ATPase α1 subunit protein contents in triad fractions from aged rats, with no apparent age-related changes in cholesterol levels or RyR1, gp91^phox^ and p47phox protein contents. We subsequently discuss the possible implications of these findings for skeletal muscle function in young and aged animals.

### Treatment with MβCD suppresses depolarization-induced Ca^2+^ transients

As reported previously, single skeletal muscle fibers from fetal mice treated with MβCD display defective E–C coupling but retain normal voltage dependence and exhibit similar action potentials as control fibers (Pouvreau et al., 2004). Our results in FDB fibers from adult mice indicate that MβCD partially removed cholesterol from these fibers, in agreement with our previous report (Llanos et al., 2015), suppressed Ca^2+^ transients in most fibers or significantly reduced their amplitude, and altered the distribution of both caveolin-3 and Cav1.1. Our determinations of Fluo-4 fluorescence suggest that MβCD treatment induced a moderate increase (≤2-fold) in myoplasmic resting [Ca^2+^] and preserved caffeine-induced Ca^2+^ release. Altogether, results from previous studies combined with the present findings suggest that partial cholesterol removal affects T-tubule components, such as Cav1.1, which participate directly in the E–C coupling process. In fact, we found that MβCD treatment caused significant disarray in the regular pattern of Cav1.1 distribution. This alteration may underlie the selective and marked reduction in Cav1.1-mediated currents produced by MβCD, since a previous study indicates that membrane cholesterol removal from skeletal fibers from fetal mice specifically targets Cav1.1 channel function since T-type Ca^2+^ currents remain unaltered (Pouvreau et al., 2004). Of note, statin treatment to inhibit cholesterol synthesis (Istvan and Deisenhofer, 2001) produces T-tubule structural abnormalities in human patients (Voigt et al., 2013). A decrease in T-tubule cholesterol content may contribute to the skeletal muscle dysfunction described in patients undergoing statin therapy; in fact, as many as 25% of statin users who exercise may experience defective skeletal muscle function (Dirks and Jones, 2006). There is no information, however, regarding T-tubule cholesterol levels in these subjects. In addition, cholesterol removal may affect caveolin-3 distribution and function, in particular its role in E–C coupling, as discussed below.

### Association of caveolin-3 with T-tubules and DRM fractions

Previous studies associated caveolin-3 with the T-tubule system only during development (Parton et al., 1997), and reported that in adult skeletal muscle caveolin-3 was exclusively present in association with the sarcolemmal membrane (Parton et al., 1997). Moreover, other studies described caveolin-3 in association with the SR membrane (Li et al., 2006). Our results in three different mammalian species—mice, rabbit and rat—show conclusively that in adult mammalian skeletal muscle this protein is associated to T-tubule and not to SR membranes, suggesting an evolutionary conserved role for caveolin-3 in T-tubule system development and preservation of structural integrity in adult muscle.

Here, we show that DRM fractions from either T-tubules or triads are highly enriched in cholesterol and caveolin-3 with respect to the original membrane fractions, and also contain Cav1.1. Our results complement previous studies showing the presence of both caveolin-3 and Cav1.2 in DRM obtained from cardiac tissue (Balijepalli et al., 2006). EM images of DRM obtained from triads revealed the presence of membrane-associated electron dense material, suggesting that in addition to caveolin-3 and Cav1.1, other proteins of the E–C coupling complex remain associated with DRM fractions. In fact, we found that DRM fractions from triads migrated at higher sucrose densities than DRM from T-tubules, indicating higher protein to lipid ratios in the former fractions. Our results also reveal that cholesterol extraction from T-tubules and triads with MβCD produced concurrent caveolin-3 extraction to the supernatants only in isolated T-tubules. Previous work showed that caveolin-3 interacts both with Cav1.1 (Couchoux et al., 2011) and RyR1 (Whiteley et al., 2012); the persistence of this T-tubule/SR protein complex in triads may explain why removing cholesterol did not extract caveolin-3 from these fractions.

### Caveolin-3 and skeletal muscle function

The levels of caveolin-3 are critical for the correct functioning of skeletal muscle, because changes in the expression of this protein produce pathological phenomena (Galbiati et al., 2000; Hagiwara et al., 2000). Caveolin-3 reduction in skeletal muscle myotubes from mice induces the opening of non-selective mechano-sensitive ion channels while caveolin-3 overexpression produces a small decrease in mechano-sensitive currents, suggesting that normal caveolin-3 expression contributes to protection of the sarcolemma from mechanical damage (Huang et al., 2013). Skeletal muscle also expresses caveolin-1 (Kawabe et al., 2001; Li et al., 2006); however, caveolin-1 does not restore completely the function of caveolin-3, since caveolin-3 knockout mice present T-tubule system abnormalities (Galbiati et al., 2001) and exhibit muscle degeneration (Hagiwara et al., 2000). Interestingly, mice null for caveolin-3 and caveolin-1 develop severe heart disease (Park et al., 2002), while caveolin-3 over expression induces a Duchenne-like phenotype in mice (Aravamudan et al., 2003). Furthermore, mice null for caveolin-3 and caveolin-1 display insulin resistance, glucose intolerance and decreased insulin-induced glucose uptake; re-expression of these proteins reverses these conditions (Capozza et al., 2005). The insulin receptor is unstable in caveolin-3 null mice; insulin binding induces its degradation suggesting that caveolin-3 stabilizes the insulin receptor (Capozza et al., 2005). Of note, caveolin-3 reduction in cardiac muscle cells produces severe alterations in cardiac function, characterized by considerable tissue degeneration, fibrosis, decreased cardiac function, and decreased activity of nitric oxide synthase (Aravamudan et al., 2003). Altogether, these reports suggest that alterations in caveolin-3 levels induce pathological conditions in skeletal and cardiac muscle.

Recent studies reported that caveolin-3 interacts with Cav1.1 (Couchoux et al., 2011) and RyR1 (Whiteley et al., 2012), suggesting that the two Ca^2+^ channels directly involved in E–C coupling establish functional links with caveolin-3. Cholesterol removal, which affects the distribution of both caveolin-3 and Cav1.1, may affect these functional connections giving rise to the defective E–C coupling responses reported in amphibian (Launikonis and Stephenson, 2001), fetal (Pouvreau et al., 2004) and adult rat skeletal muscle, where we found that partial cholesterol extraction inhibited Ca^2+^ transients. These effects are similar to the reported effects of aging on E–C coupling (Booth et al., 1994; Wang et al., 2000). Early studies indicated that rat skeletal muscle from 12-month-old rats had higher caveolin content than muscle from 3-month-old rats; however, whether this increase reflects augmented expression of caveolin-3, caveolin-1, or both was not evaluated (Munoz et al., 1996). Other early studies suggested that aging produces uncoupling between Cav1.1 and RyR1, with the consequent muscle weakness (Renganathan et al., 1997). Our results indicate that triad fractions from skeletal muscle of 24-month-old rats had 2-fold lower caveolin-3 protein levels than triads from young rats. As described above, caveolin-3 interacts with Cav1.1 and RyR1, forming a supra-molecular complex. Reduction in caveolin-3 protein levels during aging is likely to remodel or alter the integrity of this complex, causing E–C uncoupling. A decrease in caveolin-3 levels may also modify T-tubule architecture and T-tubule related signal transduction pathways, such as insulin-dependent glucose uptake.

Caveolin-3 binds directly to cholesterol (Murata et al., 1995) and sphingolipids (Haberkant et al., 2008). We found that DRM fraction from T-tubules or triads contained both caveolin-3 and Cav1.1; the possible association of these two proteins in cholesterol-enriched membrane regions supports the proposed role of caveolin-3 in regulating Cav1.1 function (Weiss et al., 2008). While we did not detect significant differences in cholesterol content between triads from young or aged rats, a marked reduction in caveolin-3 protein content during aging may affect the local cholesterol distribution in T-tubule membranes, altering the function of the caveolin-3-containing RyR1/Cav1.1 supra-molecular complex, and hence E–C coupling. Accordingly, it would be of interest to investigate in future studies if restoring caveolin-3 levels in the skeletal muscle of aged animals improves their defective E–C coupling.

### Changes in other triadic proteins during aging

#### RyR1 and Cav1.1

Our results in triads from skeletal muscle of 24-month-old rats confirm that RyR1 protein content does not change with rodent age (Russ et al., 2011). Previous reports suggest a drastic reduction of Cav1.1 and no significant change of RyR1 protein levels in total fractions from skeletal muscles of 30-month-old rats (O'connell et al., 2008). We obtained inconclusive results regarding changes in Cav1.1 protein levels with age, but we did not observe an age-related Cav1.1 reduction.

#### Na^+^/K^+^-ATPase

In rat skeletal muscle, aging causes muscle type-specific alterations in Na^+^/K^+^-ATPase activity and increases the expression of the enzyme α1 and β1-subunits, measured in total muscle homogenates (Sun et al., 1999). Immunohistochemistry analysis showed increased expression of the α1-subunit in white but not in red gastrocnemius muscle, which displays mainly a sarcolemmal pattern (Zhang et al., 2006). Increased expression of the Na^+^/K^+^-ATPase enzyme during aging, which we confirmed in triad fractions from aged muscle, may be relevant for muscle function during aging. Reduction of caveolin-3 content activates non-selective mechano-sensitive ion channels in skeletal muscle (Huang et al., 2013), whereas in cardiac muscle caveolin-3 regulates Kv1.5 potassium currents by modulating the number of functional channels in the membrane (Folco et al., 2004). Accordingly, decreased T-tubule caveolin-3 levels may result in increased Na^+^ and K^+^ fluxes via non-selective ion channels during aging, which if not compensated by active ion transport by the Na^+^/K^+^-ATPase would result in decreased excitability of skeletal muscle fibers, thereby contributing to contractile fatigue (Welle, 2002). Therefore, the increased Na^+^/K^+^-ATPase protein levels displayed by triads from aged rat muscle may reflect an adaptation mechanism to compensate for the increment of ionic T-tubule membrane permeability induced by the low caveolin-3 levels observed in aged muscle.

#### GAPDH

In addition to decreased caveolin-3 content, triads from aged rats displayed significant reductions in GAPDH protein content. Our results agree with previous reports showing that aged skeletal muscles display decreased levels of GAPDH mRNA (Touchberry et al., 2006) and protein (Vigelso et al., 2015). In skeletal muscle, GAPDH binds to RyR1, Cav1.1 and the T-tubule membrane (Brandt et al., 1990), forming a complex that may functionally couple glycolysis with SERCA-mediated Ca^2+^ transport into the SR (Xu et al., 1995). SR-associated glycolytic enzymes may produce ATP locally, resulting in modifications of myoplasmic Ca^2+^ signals, either via SERCA-mediated Ca^2+^ uptake into the SR and/or via RyR1-mediated Ca^2+^ release, because ATP is a well-characterized physiological RyR agonist (Fill and Copello, 2002; Bull et al., 2007). The higher Na^+^/K^+^-ATPase levels displayed by triads from aged rats, combined with their reduced GAPDH content, suggest that aged muscle has a reduced capacity to generate ATP locally at the triads, which may result in inhibition of SR Ca^2+^ uptake and reduced RyR1 activity. We propose that these combined factors contribute to disrupt normal Ca^2+^ signaling, thereby reducing muscle contractility in aged skeletal muscle. Future studies should address this proposal.

#### NOX

Skeletal muscle activity increases ROS production (Reid et al., 1992; Borzone et al., 1994). In several tissues, the diverse NOX isoforms contribute to ROS generation (Bedard and Krause, 2007). Skeletal muscle expresses NOX4 mRNA as well as the mRNA for the gp91^phox^ subunit of the NOX2 isoform (Cheng et al., 2001; Shiose et al., 2001), in addition to expressing proteins of the NOX2 complex (Javeshghani et al., 2002), but the role of NOX in activity–dependent ROS production in skeletal muscle remains inconclusive. For instance, NOX-mediated ROS production in diaphragm muscle does not contribute to the ROS increase produced by heat stress (Zuo et al., 2003). Nonetheless, skeletal muscle T-tubules express several NOX2 subunits and NOX2-generated ROS stimulate SR Ca^2+^ release, presumably as a result of NOX2-induced RyR1 redox modifications (Hidalgo et al., 2006). Recent studies using the ROS-sensitive probe rhoGFP indicate that contraction and stretching of skeletal muscle both activate NOX (Pal et al., 2013). We found that triads from young or aged rats contained similar p47^phox^ and gp91^phox^ protein levels and comparable NOX activities. Our results strongly suggest that NOX-generated ROS do not contribute to the increased resting ROS levels of aged muscle; however, we cannot rule out differential effects of exercise on NOX-dependent ROS production as a function of age. It has been proposed that mitochondrial and NOX are possible ROS sources in aged skeletal muscle (Bejma and Ji, 1999). Our results support a predominant mitochondrial origin for the reported increase in resting ROS production in skeletal muscle (Vasilaki et al., 2006; Umanskaya et al., 2014), which results in increased RyR1 oxidation (Andersson et al., 2011).

## Concluding remarks

In this work, we show that partial cholesterol extraction from single skeletal fibers isolated from mice adult skeletal muscle eliminated or significantly reduced electrically evoked Ca^2+^ transients, without affecting membrane permeability or causing SR calcium depletion. We propose that partial cholesterol removal impairs E–C coupling by modifying the interactions of the cholesterol-associated protein caveolin-3 with RyR1 and Cav1.1. In support of this proposal, we found caveolin-3 associated with the cholesterol-rich T-tubule membranes, but not with SR membranes; moreover, caveolin-3 is present together with Cav1.1 in cholesterol-enriched DRM fractions from T-tubules or triads. In addition, we observed significantly decreased caveolin-3 and GAPDH and increased Na^+^/K^+^-ATPase levels in triads from aged rats, but we did not detect changes in cholesterol or RyR1 protein levels. These findings suggest that age-related changes in triadic proteins, and in particular the decrease in caveolin-3 protein content, are likely to alter T-tubule related signal transduction pathways and to produce defective E–C coupling of aged skeletal muscle. Our results contribute to the field of muscle physiology by suggesting that cholesterol and caveolin-3 are important for the normal E–C coupling process, and that alterations in their levels contribute to the defective function of aged skeletal muscle.

## Author contributions

GB and PL performed experiments, analyzed data, and contributed to manuscript writing. PB, CC and AR performed experiments and analyzed data. GS performed experiments, analyzed data and critically reviewed the manuscript. JH analyzed data, contributed to the discussion of results and critically reviewed the manuscript. AQ and CH supervised the study, analyzed data and provided funding. CH wrote the manuscript and is the guarantor of this work and, as such, had full access to all the data in the study and takes responsibility for the integrity of the data and the accuracy of the data analysis.

### Conflict of interest statement

The authors declare that the research was conducted in the absence of any commercial or financial relationships that could be construed as a potential conflict of interest.

## Abbreviations

Cav1.1: Voltage-dependent Ca^2+^ channels
Ca^2+^: Calcium
DHPR: Dihydropyridine receptor
DRM: Detergent resistant membranes
E–C coupling: excitation–contraction coupling
FDB: Flexor digitorium brevis
GAPDH: Glyceraldehyde 3-phosphate dehydrogenase
HRS: Heavy sarcoplasmic reticulum
LSR: Light reticulum
MβCD: Methyl-β-cyclodextrin
NOX: NADPH-oxidase
RyR1: Type-1 ryanodine receptor
SR: Sarcoplasmic reticulum
T-tubules: Transverse tubules.
